# Covalent Organic Framework Membranes for Ion Separation: A Review

**DOI:** 10.3390/membranes15070211

**Published:** 2025-07-15

**Authors:** Yutong Lou, Zhanyong Wang, Wanbei Yang, Shuchen Lang, Jiaxing Fan, Qiaomei Ke, Rui Wang, Zhen Zhang, Wentao Chen, Jian Xue

**Affiliations:** 1 Guangdong Provincial Key Lab of Green Chemical Product Technology, School of Chemistry and Chemical Engineering, South China University of Technology, Guangzhou 510640, China; ce_yutonglou@mail.scut.edu.cn (Y.L.); 202230283219@mail.scut.edu.cn (W.Y.); celangshuchen@mail.scut.edu.cn (S.L.); 202330370691@mail.scut.edu.cn (J.F.); 202230281024@mail.scut.edu.cn (Q.K.); 2 Environmental Radiation Monitoring and Nuclear Emergency Response Technical Support Center of Guangdong Province, Guangzhou 510300, China; gdsthjt_wangzhanyong@gd.gov.cn (Z.W.); gdsthjt_zhangzhen@gd.gov.cn (Z.Z.); gdsthjt_chenwentao2@gd.gov.cn (W.C.)

**Keywords:** covalent organic frameworks, membranes, ion separation, permeability, selectivity

## Abstract

Covalent organic framework (COF) membranes have garnered significant attention in ion separation due to their high surface area, tunable pore size, excellent stability, and diverse functional groups. Over the past decade, various synthesis methods, such as solvothermal synthesis, interfacial synthesis, microwave-assisted solvothermal synthesis, and in situ growth, have been developed to fabricate COF membranes. COF membranes have demonstrated remarkable ion separation performance in different separation processes driven by pressure, electric field, and vapor pressure difference, showing great potential in a wide range of applications. Nevertheless, challenges in the synthesis and application of COF membranes still remain, requiring further research to fully realize their potential in ion separation. This review critically examines the development of COF membranes, from synthesis methods to ion separation applications. We evaluate the advantages and limitations of various synthesis techniques and systematically summarize COF membrane performance based on separation driving forces. Finally, we present a critical analysis of current challenges and offer perspectives on promising future research directions for advancing COF membrane technology in separation.

## 1. Introduction

In today’s era, with the acceleration of global industrialization and the continuous growth of population, the demand for clean water resources and partial ion recycling is becoming increasingly urgent [1,2]. Ionic separation technology plays a core role in meeting these needs, such as seawater desalination [3] and heavy metal recovery [4]. Typically, traditional separation techniques, including distillation, crystallization, and condensation, are energy-intensive processes [5], highlighting the need for efficient ion separation technology to address global water and energy shortages.

Membrane separation technology has become a research hotspot in the field of ion separation because of its unique advantages of low energy consumption, simple operation, high efficiency, and environmental friendliness [6,7]. The membrane separation processes that have been developed include microfiltration (MF), ultrafiltration (UF), nanofiltration (NF), reverse osmosis (RO), electrodialysis (ED), capacitive deionization (CDI), membrane distillation (MD), and pervaporation (PV) [8,9,10,11]. In addition, membranes with customizable ion selectivity can meet diverse needs. For instance, targeted lithium recovery from brines may be achieved with ion-selective membranes, thereby meeting the growing demands of Li battery production [12].

Nonetheless, owing to the limitation of the trade-off between permeability and selectivity of traditional polymer membranes, many prospects for membrane separation technology have not been realized yet [6]. Among numerous membrane materials, the covalent organic framework (COF) has attracted much attention due to its unique structure and properties [13]. The COF is a class of crystalline polymer materials with highly ordered porous structures [14] and is formed by organic building units connected by strong covalent bonds to form one-dimensional (1D), two-dimensional (2D), or three-dimensional (3D) porous networks. The pore size of COFs can be precisely controlled by selecting different building units and synthesis methods [15,16], thereby achieving size exclusion of specific ions. In addition, COFs have abundant chemical functional groups, such as hydroxyl groups, sulfonic acid groups, carboxyl groups, and quaternary ammonium groups, which can be introduced through monomer design and pore wall post-modification to enhance subject–object interaction, like electrostatic interaction and bonding interaction for specific ions [17]. At the same time, COF materials have good chemical stability and mechanical strength [18] and can operate stably for a long time under different operating conditions.

This review systematically summarizes the latest research progress of COFs membranes in the field of ion separation; deeply explores their separation mechanisms, preparation methods, and application examples; focuses on analyzing the roles of membrane pore size, surface charge, and interaction forces with separated substances in ion separation; and looks forward to the development direction of COFs membranes in future ion separation technologies.

## 2. The Synthesis of COF Membranes

### 2.1. Solvothermal Synthesis

Most COF materials are synthesized via solvothermal synthesis, primarily for producing COF powders [19,20,21]. The typical process involves placing monomers in a Pyrex tube with appropriate organic solvents and a catalyst, removing air from the system through freeze–pump–thaw cycles, and finally sealing the tube at high temperature. The sealed tube is then heated to a specific temperature, triggering a complex polymerization and crystallization procedure over 1–10 days, followed by purification to yield COF powder [22,23,24]. Laemont et al. [25] successfully synthesized highly crystalline and porous imine-linked COFs via a mild and scalable solvothermal method. Their work reveals that long-chain alkyl alcohols enhance COF nanosheet aggregation and ordered stacking, thereby improving crystallinity and pore robustness.

**Figure 1:**
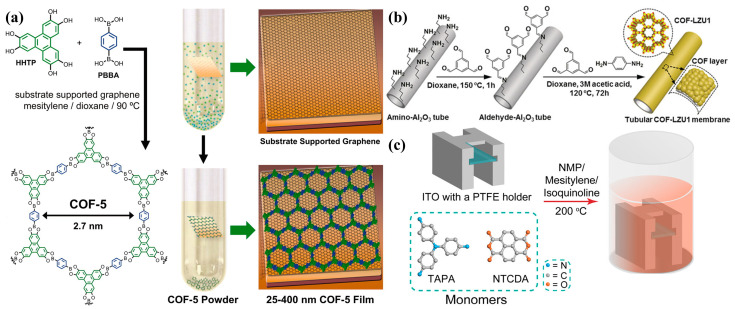
(**a**) Solvothermal condensation of HHTP and PBBA in the presence of a substrate-supported SLG surface provides COF-5 as both a film on the graphene surface, as well as a powder precipitated at the bottom of the reaction vessel (adapted from [26]). (**b**) Synthesis of tubular COF-LZU1 membranes (adapted from [27]). (**c**) Schematic diagram of the solvothermal synthesis of a PI-NT COF film on an indium tin oxide-coated glass substrate (adapted from [28]).

Due to continuous research, solvothermal synthesis is no longer limited to the preparation of COF powder and can also be used to synthesize COF membranes. Colson et al. [26] successfully synthesized vertically oriented 2D COF thin films (like COF-5) on single-layer graphene (SLG) substrates via solvothermal methods (Figure 1a). Fan et al. [27] successfully prepared a continuous 2D imine-linked COF-LZU1 membrane with a thickness of only 400 nm on the surface of alumina tubes via in situ solvothermal synthesis (Figure 1b). The porous substrates were modified by 3-aminopropyltriethoxysilane (APTES) and 1,3,5-triformylbenzene (TFB) to enhance adhesion between the COF layer and substrates. Sun et al. [28] successfully fabricated ordered donor–acceptor (D-A) structured 2D polyimide (PI-NT) COF thin films on indium tin oxide (ITO)-coated glass substrates via a solvothermal method (Figure 1c). By optimizing the monomer concentration and reaction conditions, they obtained highly crystalline PI-NT COF films with good orientation preference and low surface roughness.

However, there are still some drawbacks to the solvothermal method for preparing COF membranes. This method usually requires toxic, high-boiling-point organic solvents, complicating post-treatment and raising environmental concerns. This process also requires high temperature and pressure conditions, leading to high energy consumption, specialized equipment needs, and safety risks. Solvothermal synthesis is commonly used for the synthesis of COF powder; the resulting powder is prone to accumulating unevenly, making it difficult to form uniform, defect-free membranes capable of withstanding dynamic stresses during the separation process. In addition, the long reaction time hinders rapid membrane formation, and equipment limitations restrict large-area continuous preparation [29].

### 2.2. Interfacial Synthesis

Interfacial synthesis is a strategy used for achieving the controllable growth of COF membranes by regulating reaction conditions at two-phase interfaces, such as liquid–liquid, gas–liquid, or solid–liquid interfaces [30,31,32,33,34,35,36,37,38].

**Figure 2:**
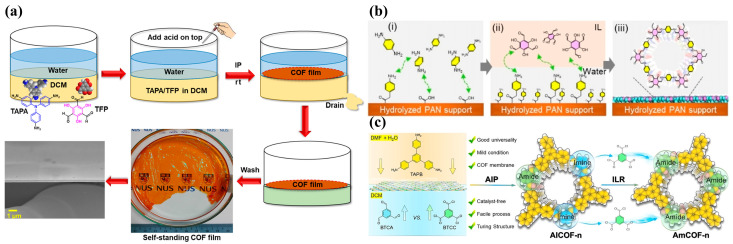
(**a**) Fabrication process of the self-standing COF films (adapted from [39]). (**b**) Schematic illustration of interfacial polymerization of TpPa COF layer at the ionic liquid-H_2_O (IL–H_2_O) interface. (**i**) Anchoring of Pa monomers on the surface of hydrolyzed PAN support via electrostatic interaction. (**ii**) Interfacial polymerization of 1,3,5-triformylphloroglucinol (Tp) and p-phenylenediamine (Pa) at the ILs–H_2_O interface. (**iii**) Formation of TpPa COF layer on the surface of hydrolyzed PAN support (adapted from [40]). (**c**) Synthetic strategy and procedure of the representative AmCOF-1 membrane (adapted from [41]).

Liu et al. [39] introduced a simple and effective strategy for constructing self-supporting and flexible COF membranes through liquid–liquid interface-confined reactions at room temperature and atmospheric pressure (Figure 2a). For the highly ordered honeycomb lattice, COF membranes exhibit high solvent permeability. Pan et al. [40] reported a new scraping-assisted interface aggregation (SAIP) technique for preparing scalable and uniform TpPa-COF membranes (Figure 2b). This method uses non-toxic and low-volatility ionic liquids as organic phases to replace traditional organic solvents, synthesizing TpPa-COF layers on a porous supporting membrane. It produces large-area continuous COF membranes with a COF layer thickness of 78 nm in just 2 min, achieving high permeability and excellent antibiotic desalination efficiency. Fang et al. [41] proposed an autocatalytic interfacial polymerization strategy to synthesize highly crystalline amide and imine bilinker COF (AICOF) and irreversibly amide-linked COF (AmCOF) membranes without additional catalysts (Figure 2c). The AmCOF-1 membrane achieved an H_2_O_2_ production rate of 4353 µmol g^−1^ h^−1^ with excellent stability in the photocatalytic synthesis of H_2_O_2_.

As an important method for preparing COF membranes, interfacial synthesis offers advantages in material structure control and separation performance improvement, yet it has shortcomings. This approach usually requires precise control of the fabrication parameters, such as interfacial tension of the two-phase solvents, monomer concentration, synthesis temperature, and reaction time, as minor deviations can compromise membrane quality. The resulting COF membranes synthesized are often nanoscale-thin, mechanically unstable, and susceptible to cracking or peeling during transfer or use. Additionally, the rapid reaction rate may lead to incomplete crystallization, resulting in low crystallinity or amorphous structure, which impairs the separation ability of COF membranes.

### 2.3. Microwave-Assisted Solvothermal Synthesis

Microwave-assisted solvothermal synthesis combines the solvothermal method and microwave heating. Microwaves can penetrate media and directly interact with polar molecules, rapidly generating heat through molecular dipole polarization and ion conduction, enabling faster heating and more uniform “body heating” [14,20,42].

Campbell et al. [43] first employed a microwave-assisted solvothermal method for synthesizing COFs, reducing the reaction time to 20 min—a more than 200-fold speed increase. Using COF-5 and COF-102 as models, efficient synthesis was achieved in both sealed and open containers. The prepared COF-5 and COF-102 exhibited specific surface areas of 2019 m^2^ g^−1^ and 2926 m^2^ g^−1^, respectively, outperforming the conventional solvothermal method. This method eliminates high-pressure requirements, achieves high yields (up to 95% for COF-5 in an open container), and works for 2D and 3D COFs.

**Figure 3:**
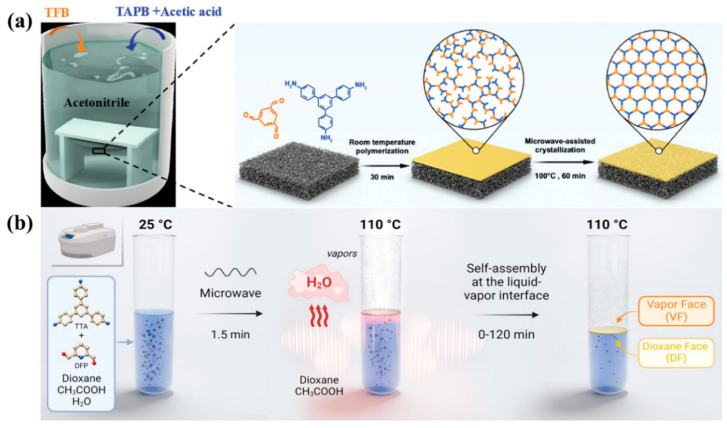
(**a**) Schematic illustration of the fabrication process of 1,3,5-triformylbenzene tris(4-aminophenyl)amine (TFB-TAPB) membrane (adapted from [45]). (**b**) Schematic representation of the membrane formation inside the microwave vessel. The self-standing TTA-DFP-COF membrane is formed due to the confinement of the polymerization of the aldehyde and amine monomers at the interface between dioxane and water vapors under microwave irradiation (300 W) (adapted from [46]).

Hao et al. [44] presented a novel synthesis method for synthesizing a COF-5 membrane on a functionalized α-Al_2_O_3_ ceramic support. The porous α-Al_2_O_3_ support was surface-modified using 3-aminopropyltriethoxysilane (APTES) and 4-formylphenylboronic acid (FPBA) as covalent linkers, and then the COF-5 membrane was successfully grown via microwave irradiation. The characterization confirmed the formation of a continuous and compact COF-5 layer (~1 μm thick) on the modified support, while unmodified supports yielded only discrete COF-5 particles. Xu et al. [45] presented a microwave-assisted two-step strategy for fabricating highly crystalline and robust COF membranes, which exhibited a highly ordered pore structure, achieving exceptional organic solvent nanofiltration performance (Figure 3a). Benyettou et al. [46] presented a dual-faced COF membrane (TTA-DFP-COF) synthesized via a rapid microwave-assisted liquid-vapor interfacial self-assembly method (Figure 3b). The membrane thickness is tunable by adjusting the reaction time, and surface properties can be controlled through differential chemical groups.

However, there are still shortcomings in the microwave-assisted solvothermal method. Microwave heating relies on the dielectric loss of polar solvents to generate heat, so polar solvents must be used for effective heating, which limits the diversity of solvent systems and may affect the chemical stability and functional regulation of COF membranes. In addition, some polar solvents are toxic or difficult to recover, increasing the complexity of subsequent processing. For high-boiling-point organic solvents, reduced-pressure distillation is typically employed for purification, followed by dehydration via molecular sieves and gas chromatography verification before reintroduction to synthesis. For mixed solvents, separation techniques such as liquid separation or salting-out must first isolate organic components prior to distillation. Regarding toxic or difficult-to-treat solvents like halogenated solvents, strict transfer to specialized hazardous waste disposal agencies is mandatory for compliant treatment through incineration or solidified landfill.

Microwave heating accelerates the nucleation process but may result in insufficient crystal growth, leading to amorphous regions or microporous structural defects. Although microwave methods perform well on a laboratory scale, their industrial application faces challenges due to high equipment costs and process adaptability issues.

### 2.4. In Situ Growth

In situ growth is a technique used for directly synthesizing COF membranes on the substrate surface [47,48,49] by adjusting reaction conditions, enabling COFs to self-assemble into a continuous separation layer supported by the substrate. This method combines the high porosity, adjustable pore size, and chemical functionalization properties of COF materials while utilizing the structural advantages of the substrate.

**Figure 4:**
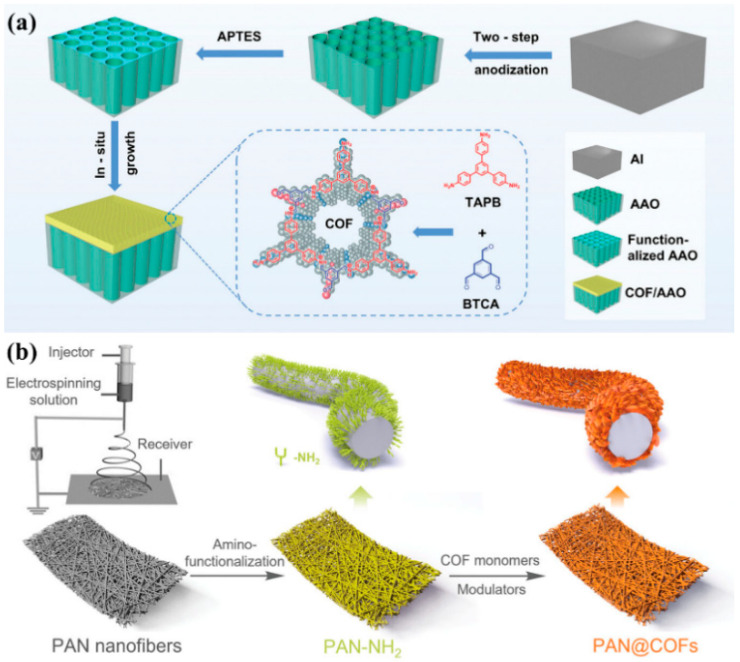
(**a**) Schematic illustration of the fabrication process of COF/AAO membrane (adapted from [50]). (**b**) Schematic representation of the in situ growth of COF on amino-functionalized PAN nanofibers prepared by electrospinning (adapted from [51]).

Chen et al. [50] developed an anion-selective nanofluidic membrane by growing an imine-bridged COF on anodic aluminum oxide (AAO) substrate at room temperature (Figure 4a). The membrane’s positively charged surface, formed by protonated imine and residual amino groups in COF under neutral pH, preferentially transports anions and exhibits a significant ion current rectification effect due to the asymmetric charge and structural characteristics of COF membranes. This design effectively reduces ion concentration polarization while improving ion selectivity and permeability. The experimental results showed a power density output of 17.95 W m^−2^ under a 500-fold salinity gradient—8 times higher than non-selective AAO membranes—with excellent long-term stability. Zhao et al. [51] prepared vertically aligned COF nanoplates on polyacrylonitrile (PAN) nanofiber substrates via in situ growth (Figure 4b). By functionalizing PAN fibers with amine groups as nucleation sites and employing a reversible polycondensation–termination approach, a flexible PAN@COF composite membrane with a high surface area, efficient mass transport, and hierarchical porosity was developed. The membrane demonstrated exceptional adsorption performance for the antibiotic ofloxacin (OFX), achieving a capacity of ~236 mg g^−1^ and 98% removal efficiency. The method’s versatility was proven by synthesizing diverse COF variants using different amine linkers. The membrane’s mechanical robustness, chemical stability, and multi-scale porous structure highlight its potential for advanced water purification and pollutant-adsorption applications.

However, the in situ growth synthesis process is highly sensitive to reaction conditions such as temperature, concentration, and time, which results in poor controllability of membrane thickness, uniformity, and crystallinity. The surface properties of the substrate material have a significant impact on COF membrane formation, limiting the flexibility of substrate selection. In situ growth usually requires high temperatures or inert gas protection, which has high requirements for equipment and entails high energy consumption. In addition, the synthesis cycle is lengthy, repeatability is poor, and some systems require harsh conditions or toxic solvents, which increases the environmental burden and the difficulty of large-scale preparation. The factors mentioned above collectively constrain the application of COF membranes.

### 2.5. Comparison of the Synthesis Methods

Table 1 compares the advantages and disadvantages of the four widely used COF membrane synthesis methods mentioned above.

The conventional solvothermal method is prone to synthesizing non-uniform and defective COF membranes, which significantly impact ion selectivity and the continuity of ion transport pathways. Ion selectivity highly depends on the matching between pore size and the hydrated radius of target ions. A uniform pore size distribution significantly enhances the membrane’s selectivity and stability, reducing performance fluctuations caused by defects, but a non-uniform pore size distribution diminishes the permeability difference between different ion valences [55,56]. Defects like pore channel blockages, lattice imperfections, or non-continuous stacking hinder rapid ion migration and increase mass transport resistance [57]. Interfacial synthesis can solve this problem and prepare uniform COF membranes.

However, interfacial synthesis often yields COF membranes with moderate crystallinity, negatively impacting their ion separation performance. These membranes, marked by lattice defects or discontinuous stacking, have broad pore size distributions that poorly match the hydrated radii of target ions, compromising size-based sieving [58]. High crystallinity endows COF membranes with rigid and ordered pore channel structures, ensuring the precision of molecular sieving effects, and low crystallinity leads to an uneven surface charge distribution and reduces the ability to retain multivalent ions [59,60].

It is worth noting that both solvothermal synthesis and interfacial synthesis rely on organic solvents. Solvothermal synthesis commonly uses toxic organic compounds such as DMF and DMSO, which easily volatilize to pollute the atmosphere or seep into groundwater. Interfacial synthesis employs non-degradable organic solvents like n-hexane and dichloromethane to form interfaces. Therefore, it is important to recover the organic solvents: solvothermal synthesis can use reduced-pressure distillation or rectification to recover the solvents; interfacial synthesis can separate and recycle the organic phase after phase separation, reducing fresh solvent consumption and contamination.

Due to the disadvantages mentioned above, more in-depth research will be required in the future to refine and improve the synthesis methods.

## 3. Separation Mechanisms in COF Membranes

COF membranes enable precise ion separation through a hierarchical interplay of physical confinement, electrostatic forces, and chemical affinity [61]. Unlike conventional polymer membranes constrained by the permeability–selectivity trade-off, COFs, which have evenly sized nanopores, could improve both permeance and selectivity [62]. On the one hand, the ultra-high porosity and achievable smaller membrane thickness could significantly improve the permeability of membranes. On the other hand, the uniform and adjustable pore size achieves excellent selectivity. When the channel size is precisely engineered between the dynamic diameters of two kinds of ions, the smaller one can pass through easily, while the larger one will be rejected. Therefore, the pore size of membranes plays an important role in separation.

In addition to size sieving, the interaction between ions and the membrane, like electrostatic forces, could offer a higher repulsive force, especially for discriminating between different or even identical charged ions [63]. When ions approach the surface of membrane pores with fixed charges, they are strongly attracted or repelled by charges: ions with opposite charges to the membrane pores are attracted by static electricity and are easily enriched into the pores, and ions with the same charge as the membrane pores are repelled by static electricity, making it difficult for them to enter the pores. This sieving effect is governed by Coulomb’s law, suggesting that the electrostatic interaction strength depends jointly on the ion valence state and membrane charge density. For instance, Zheng et al. [64] modified COF membranes with a cationic polymer, resulting in a rejection rate of 98.5% and 97.1% to Cr^3+^ and Mg^2+^. This effect is essentially a direct regulation of ion transport direction by long-range electrostatic forces.

For ions that cannot be sieved well based on the above mechanism, chemical affinity becomes the key to improving separation efficiency. Certain functional groups (such as oligoether chains and amino groups) [55,65,66] in COF membranes can form coordination bonds, hydrogen bonds, or electrostatic forces with particular ions. This chemical affinity enables COF membranes to selectively adsorb and transport certain ions. When an ion solution passes through a COF membrane, ions with higher chemical affinity are more likely to be captured or bound by the functional groups in the membrane, which slows down their transport or even retains them, while ions with weaker interactions pass through more easily. For example, owing to carbonyl group rings, the potassium ion channel KcsA demonstrates extraordinary selectivity for K over Na ions (1000:1), even in environments dominated by Na ions [67]. Fully utilizing the chemical affinity between the membrane and ions could greatly enhance the separation capability.

## 4. Ion Separation and Application

In membrane separation technology, the driving forces helping ions to pass through the membrane mainly include pressure difference, electric field force, and vapor pressure difference. The following section provides a detailed explanation of the separation principles and application of COF membranes under three different driving forces.

### 4.1. Separation Driven by Pressure

When high pressure is applied to one side of a membrane, the mechanical driving force generated by the pressure differential promotes ion migration by overcoming the membrane’s resistance. Under pressure-driven conditions, solvent molecules and ions pass through the membrane pores, where the pore size and porous structure play a crucial role. The membranes with larger pores, like MF and UF membranes, primarily restrict the transport of bulky particles or macromolecules, while ions face little resistance during the separation process driven by the pressure gradient [68]. Conversely, for the membranes with smaller pore dimensions, such as NF and reverse osmosis RO membranes, ion transport becomes constrained by pore walls, leading to increased collisions and frictional interactions. In these cases, additional factors, including membrane hydrophilicity and charge distribution, significantly influence ion transport behavior [69,70].

**Figure 5:**
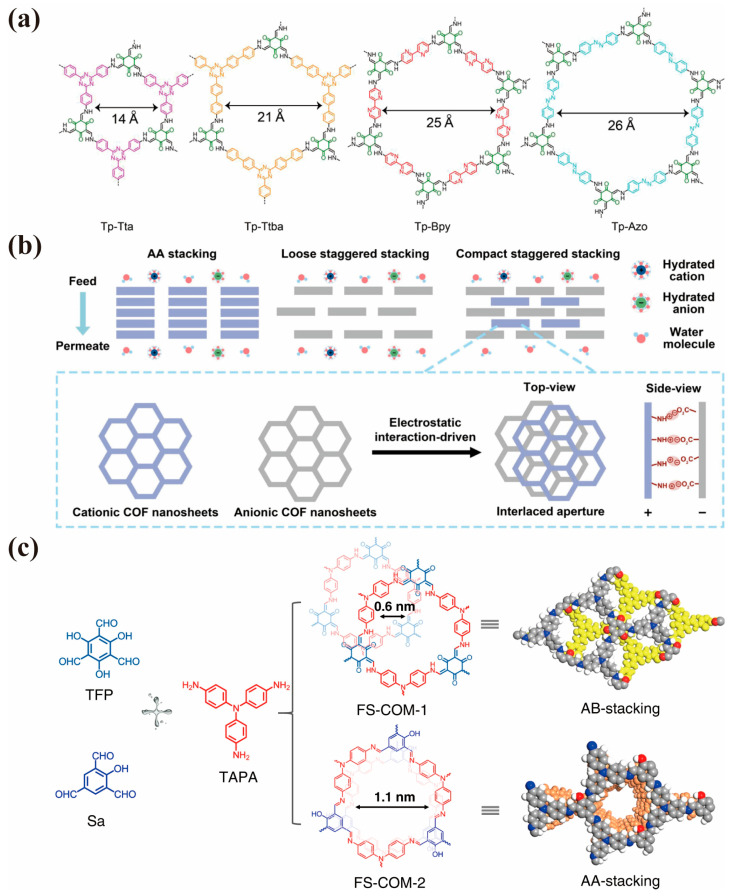
(**a**) Chemdraw structures of four COFs (including 1,3,5−triformylphloroglucinol 2,2′−bipyridine−5,5′−diamine (Tp-Bpy), 1,3,5−triformylphloroglucinol 4,4′−azodianiline (Tp−Azo), 1,3,5−triformylphloroglucinol 4,4′,4″−(1,3,5−triazine−2,4,6−triyl) trianiline (Tp−Tta) and 1,3,5−triformylphloroglucinol 4,4′,4″−(1,3,5−triazine−2,4,6−triyl) tris(1,1′−biphenyl) trianiline (Tp−Ttba)) used for synthesizing the thin films via interfacial crystallization process (adapted from [36]). (**b**) Schematic illustration of cationic and anionic nanosheets staggered–stacked 2D COF membranes for efficient molecular and ionic sieving (adapted from [82]). (**c**) Structures of the monomers and the as−designed two free−standing (AB− and AA−stacked) COF membranes (adapted from [84]).

COF membranes are widely used in size-based discrimination due to their ordered nanochannel, adjustable pore size, and uniform mass transfer pathways. From a topological viewpoint, COFs are divided into two- and three-dimensional. In terms of 2D COFs, according to reticular chemistry, the pore size of COFs could be adjusted solely by integrating different-sized building blocks and linkers. Dey et al. [36] adopted a bottom-up interfacial crystallization strategy to prepare four defect-free COF thin films with different pore sizes (Tp-Bpy, Tp-Azo, Tp-Tta, and Tp-Ttba) by simply changing the precursors (Figure 5a). The prepared COF membranes with larger apertures exhibited high rejection rates (>80%) for dye molecules and long-term stability. Fang et al. [71] set up diffusion cells on both sides of polyvinylidene fluoride (PVDF) substrate and utilized the unidirectional diffusion method to synthesize continuous and defect-free large-pole COF (LP-COF) separation layers on the substrate surface. Benefiting from the large pore structure of LP-COF, the membrane showed an unprecedented water permeance of ~3147 L m^−2^ h^−1^ MPa^−1^ with a high Congo red rejection (~92.6%), and stable performance over multiple filtration cycles. Beyond variable aperture 2D COFs synthesized by changing building blocks and linkers, 3D COFs also exhibit mutable pore sizes due to interpenetrating topological structure and dynamic response toward external stimuli [72,73,74,75]. Shi et al. [76] synthesized high-crystallinity 3D-OH-COF membranes with narrowed pore sizes on mesoporous crosslinked polyimide (CPI) supports via solvothermal growth. It turned out that the 3D-OH-COF membrane showed excellent rejection above 95% for dye molecules with a molecular weight > 300 g mol^−1^.

The pore size of COF is not only designed for separating large molecules, but it is also reduced to the sub-nanometer scale to separate salt ions. Only monomer design or group modification can be used to achieve a sub-nanometer pore size. Most polycrystalline 2D COFs, as mentioned above, commonly adopt an AA stack in their crystalline phase, which maximizes the attractive London dispersion interaction between the layers, similar to graphite and hexagonal boron nitride [77,78]. Typically, the alternation of interlayer stacking could significantly influence the physical and chemical characteristics, like pore size and stability. By leveraging weak interlayer forces (such as π-π interactions), the stacking mode of 2D COFs can be tuned between AA, AB, and ABC via three approaches: (i) controlled reaction parameters [79], (ii) intentional steric hindrance design [80], and (iii) applied external stimuli [81].

Using an oil–water–oil triphase interfacial polymerization approach, Wang et al. [82] alternately stacked cationic DhaTG_Cl_ and anionic TpPa-CO_2_H nanosheets on substrates to deliberately misalign the inherent in-plane nanopores, thereby reducing the effective pore size of the membranes. (Figure 5b). The resulting membranes exhibited considerable ion rejection for monovalent (77.9% for NaCl) and divalent ions (85.8% for Na_2_SO_4_), ascribed to the narrowed aperture, optimized stacking pattern, and compact staggered structure. Additionally, the experimental results also indicated that this membrane effectively rejects small charged or neutral molecules, achieving 89.5% rejection for rhodamine B (RhB) and 88.4% for tetracycline antibiotic (TC), both with solvated diameters of ~10 Å. Yang et al. [83] developed a COF-based membrane with an aperture less than 1.0 nm utilizing cellulose nanofibers (CNFs) as a shielding building block. Although the acquisition of sub-nanopores comes at the expense of pore uniformity, this work showed us that if we could use the sheltering effect of the 2D COF layer itself, we could obtain a membrane with sub-nm pores and a uniform pore distribution [84]. In 2D COF, the adjacent layers are maintained by noncovalent interactions, such as London dispersion forces and π-π stacking interactions [85], which implies that the stacking structure of COF can change when the interaction forces between neighboring layers are enhanced. There are two primary strategies for modulating the configuration of adjacent layers. One involves incorporating functional groups into monomers to enhance steric hindrance and attenuate π-π interactions [86,87]. The other reduces the interlayer spacing via external modulation, which suppresses intramolecular bond vibrations and induces stacking displacement [88]. Li et al. [84] simply increased the number of phenolic hydroxyl groups on monomers to increase the steric resistance, which successfully adjusted the stacking mode of 2D COF layers from an AA-stacked to AB-stacked mode and reduced the pore size from >1 nm to the sub-nm scale (Figure 5c). The resulting COF membranes with the AB stacking mode showed excellent rejection rates (90 to 95%) for Na_2_SO_4_ and K_2_SO_4_, accompanied by a slight decrease in flux due to the reduction in interlayer spacing.

### 4.2. Separation Driven by Electric-Field 

When an external electric field is applied, charged ions in the feed solution migrate directionally toward oppositely charged electrodes and selectively pass through the ion exchange membranes. Cation exchange membranes contain fixed negative charge groups, allowing only cations to pass, while anion exchange membranes have fixed positive-charge groups, permitting only anions. This selective permeability enables electric field-driven ion separation, forming the theoretical foundation for ED [9,89] and CDI [90,91] technologies. Although the ordered nanochannel and functionalities make COFs promising membrane materials, their pores (1–5 nm) are too large to effectively separate hydrated ions (<1 nm) according to the physical size exclusion mechanism [92]. To address this issue, researchers have incorporated functional groups into COF membrane pores to improve ion separation performances by enhancing chemical interactions with ions. For example, the charged COF membranes were prepared to achieve high ion rejection via electrostatic repulsion [55,64] Additionally, COF membranes with functional groups that possess significant affinities with ions can achieve efficient ion sieving through bonding interactions or solvation effects [93,94].

**Figure 6:**
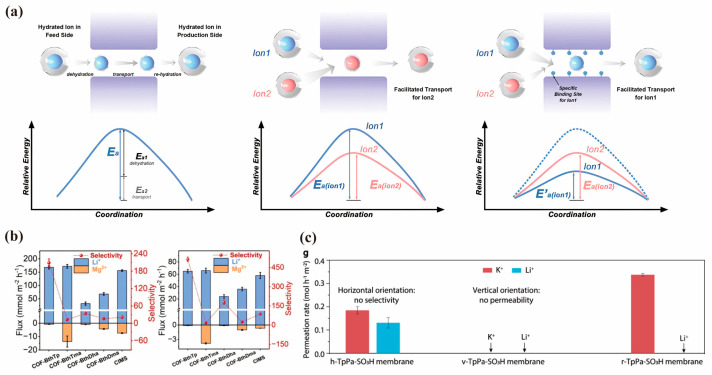
(**a**) Two energy barriers of ion transporting through sub−nanometer pores (adapted from [96]). (**b**) Investigation of transmembrane ion transport and comparative analysis of Li/Mg selectivity across different membranes under ED conditions (Left: under single−salt conditions using 0.1 M LiCl and 0.1 M MgCl_2_. Right: under binary−salt conditions with equal concentrations of 0.1 M LiCl and 0.1 M MgCl_2_) (adapted from [101]). (**c**) Permeability comparison of the tested COF materials, suggesting the importance of the narrow channels induced by the random orientation for high selectivity (adapted from [102]).

Ion transport through sub-nanometer pores is composed of two sequential steps: partitioning into the pore and intrapore diffusion [95]. Hence, the total energy barrier can be split into two parts: dehydration energy and intrapore diffusion energy [96] (Figure 6a). Zhou et al. [97] found that the former depends on pore size, while the latter hinges on the interactions between ions and the pore wall. Particularly, in the extraction of lithium from seawater or salt lakes, the separation of Li^+^ relies more on the electrostatic forces between the functional groups of COFs and ions, as the hydration diameters of competing metal ions (Mg^2+^: 8.6 Å, Na^+^: 7.2 Å, K^+^:6.6 Å) are similar to those of Li^+^ (7.6 Å). Oxygen-containing groups have exhibited substantial promise in facilitating Li^+^ transport [98,99,100]. Meng et al. [101] utilized a liquid–solid–liquid interfacial polymerization technique on polyacrylonitrile (PAN) ultrafiltration substrates, synthesizing four distinct COF membranes: COF-benzene-1,3,5-tricarbohydrazide triformylphloroglucinol (BthTp), COF-benzene-1,3,5-tricarbohydrazide 2,4,6-trimethoxybenzene-1,3,5-tricarbaldehyde (BthTma), COF-benzene-1,3,5-tricarbohydrazide 2,5-dihydroxyterephthalaldehyde (BthDha), and COF-benzene-1,3,5-tricarbohydrazide 2,5-dimethoxyterephthalaldehyde (BthDma), which have different distances and arrangements of oxygen-containing groups (carbonyl, hydroxyl, and methoxy groups) and exhibited different activation energies of Li^+^ (5.1–7.5 kJ mol^−1^) and Mg^2+^ (6.9–13.6 kJ mol^−1^). Using a test with a single salt solution and binary-salt conditions with 0.1 M LiCl and 0.1 M MgCl_2_, the resulting COF membranes with a higher density of oxygen species showed higher flux of Li as follows: COF-BthTp ≈ COF-BthTma > COF-BthDma > COF-BthDha (Figure 6b). The COF-BthTp membrane achieved a Li^+^/Mg^2+^ ideal selectivity of 209 in the electrochemical test. According to the results of molecular dynamics simulations on energy changes as ions approach and radial distribution function analysis between the oxygen atoms in the COF and the ions, Li ions encountered lower energy barriers during transmembrane migration and showed a higher peak, which reveals that Li ions occupied the binding sites with oxygen atoms. COFs on the vertical nanosheet templates tended to grow vertically. Bao et al. [102] engineered randomly oriented COF membranes by strategically harnessing the competition between intrinsic horizontal alignment and externally guided vertical orientation during synthesis. Crucially, comparative studies revealed that the horizontal orientation yielded oversized pores (1.7 nm) with no ion selectivity, whereas the vertical orientation induced excessively narrow channels that sacrificed permeability, validating a random orientation as the optimal strategy to solve the ubiquitous selectivity–permeability trade-off (Figure 6c). Under the joint action of sulfonyl groups, the resulting membrane exhibited an effective pore size of <0.32 nm and enabled selective permeation of Na+ and K+ while blocking Li+.

### 4.3. Separation Driven by Vapor-Pressure

When there is a difference in vapor pressure on both sides of the membrane, it drives solvent molecules to migrate from the high-pressure side to the low-pressure side. The selective permeability of the membrane allows only specific ions or molecules to pass, underpinning the basic principle of membrane MD [103] and PV [104,105]. MD and PV are emerging membrane-based separation technologies for liquid mixtures. MD uses a hydrophobic microporous membrane and a temperature-driven vapor pressure gradient to evaporate volatile components (e.g., water in desalination), which then condense on the permeate side. PV, however, employs dense or composite membranes and a chemical potential gradient, where components dissolve and diffuse through the membrane based on affinity, undergoing direct phase change during permeation (e.g., ethanol dehydration). MD operates at moderate temperatures (below boiling) and is suited for concentration or desalination, while PV excels at separating azeotropes or trace components (e.g., <1% water removal) at lower temperatures, making it ideal for heat-sensitive systems. Their main differences lie in phase change mechanisms.

**Figure 7:**
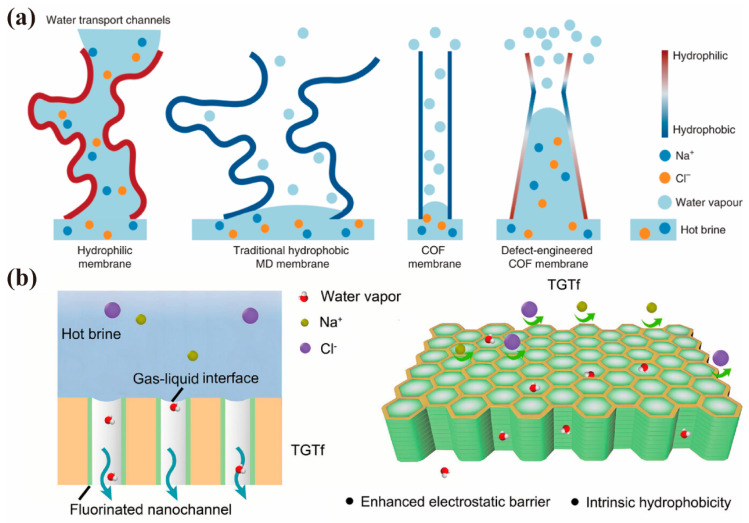
(**a**) Comparison of different water transport channels in traditional hydrophilic and hydrophobic membranes, a pristine COF membrane, and a defect−engineered COF membrane during MD processes (adapted from [108]). (**b**) Schematic representation of the MD process using the TGTf membrane, which allows for water vapor penetration while resisting wetting by liquid water (adapted from [112]).

Mass transfer in MD is controlled by three basic mechanisms: Knudsen diffusion, Poiseuille flow (viscous flow), and molecular diffusion [106]. This gives rise to three types of resistance to mass transfer: (i) Knudsen resistance (collisions of molecules with pore walls), (ii) viscous resistance (friction between molecules and pore walls), and (iii) molecule resistance (collisions among molecules). To alleviate the mass transfer resistance, shortening the diffusion path of water vapor in membrane pores is a valid solution. It is worth mentioning that nanoscale conduits and nanopores have been proven to enhance capillary evaporation [106,107]. Hence, COF membranes could serve as a suitable material for MD due to their straight channels and nano-scaled pores. Zhao et al. [108] utilized unidirectional etching to transform regular pores of COF membranes into orbitals with hydrophilic gradient and pore size gradient at liquid–solid interfaces (Figure 7a). Under the same condition, compared to PTFE membranes (75 L m^−2^ h^−1^ permeance, >21% rejection loss after 50 h), the resulting COF membranes achieved a water flux of 370 L m^−2^ h^−1^ and a salt rejection rate at 99.99% in a 100 h MD process. The vertically aligned channels in COFs deliver a minimized transfer path, enabling superior ionic sieving efficiency through unidirectional transport pathways. Membrane fouling is also a major bottleneck hindering the promotion of MD [109], driving increasing attention to the hydrophilic and hydrophobically modification of the membranes to increase their anti-fouling capability [110]. Zhao et al. [111] applied imine-based COF materials for surface modification of hydrophobic substrates, which not only increased the surface hydrophilicity but also hindered the interaction between pollutants and materials due to the dense pore structure. As a result, the COF-LZU1 modified membrane exhibited rejection rates of ≥98% for the four emerging contaminants (ibuprofen, sulfamethoxazole, DEET, and acesulfame), outperforming other MD studies. To improve anti-wetting properties, Wang et al. [112] introduced fluorine atom into COF membrane (Figure 7b) to enhance its intrinsic hydrophobicity, achieving a high permeation flux of 195 L m^−2^ h^−1^ and 99.80% salt rejection over 168 h—the longest operating time for COF membranes.

PV utilizes a chemical potential gradient to facilitate the dissolution and diffusion of specific components through the membrane based on affinity, achieving separation. The transportation of specific components through membranes involves three steps: adsorption, diffusion, and desorption [104]. The diffusion process that occurs in the second step is commonly referred to as the rate determination step based on Fick’s first law, as the first and third steps can quickly reach equilibrium [113]. It is proposed that the strong hydrophobicity and large pores of COF may enhance the diffusion of organic solvents. Wu et al. [114] fabricated COF-LZU1/PDMS mixed matrix membranes (MMMs) for the separation of n-butanol and water using PV technology, achieving a permeation flux of 2694 g m^−2^ h^−1^ and a separation factor of 38.7.

## 5. Conclusions and Outlooks

This review discusses promising potential approaches for preparing and using COF-based membranes for ion separation. Although great progress has been made in the preparation and application of COF-based membranes, some key issues remain to be addressed to improve their performance and expand their practical applications.

(1) COF membranes are primarily synthesized via solvothermal synthesis, which achieves high crystallinity but entails harsh reaction conditions and toxic solvents; interfacial synthesis, enabling rapid and uniform formation under mild conditions yet producing mechanically weak membranes; microwave-assisted solvothermal synthesis, offering fast and high-yield synthesis but restricted by solvent compatibility; and in situ growth, integrating membranes directly onto substrates for tailored functionality but requiring precise control and facing scalability challenges. Future research aims to enhance physical and chemical properties; improve convenience, eco-friendliness, and scalability in manufacturing; and expand applications in energy, catalysis, and separation fields.

(2) Most reported COF materials have pore sizes larger than 0.7 nm, and researchers have focused more on exploring the application of COF membranes in nanofiltration, with little attention paid to the development of sub-nanometer-sized COF membranes. Some studies have reported reducing pore size by alternating stacking with other materials, but disordered stacking can mask the advantage of uniform pore size in COF materials, making ion transport channels more complex and reducing mass transfer efficiency. Introducing certain functional groups into COF monomers and altering the binding sites of monomer chemical bonds to reduce pore size is a promising research direction. At the same time, with the help of external forces such as external pressure and solvent types, the π-π interaction force between COF material layers can be weakened, and the stacking method of adjacent layers can be changed to effectively reduce the pore size.

(3) In addition to traditional filtration techniques, COF membranes also have great application prospects in electrochemical-assisted and thermally assisted membrane separation technologies. The mass transfer resistance experienced by ions passing through membrane pores mainly comes from the friction and collision between ions and pores, which is further divided into the obstruction of ions by the pore size before entering the pores and the interaction force between the pore wall and ions after entering the pores. The linear pore channels of COF materials can greatly shorten the transport path of ions, accelerate mass transfer speed, and enhance permeability. At the same time, the functional groups in COF monomers can interact with compounds, such as oxygen-containing functional groups that can generate solvation interactions with metal ions, achieving selective passage of the target substance. Especially, previous studies have shown that nanoscale pores can enhance capillary evaporation and further promote thermal-assisted separation efficiency. In the future, more scholars should explore the advantages and applications of COF materials in these fields.

## Figures and Tables

**Table 1:** Comparison of four synthesis methods.

Synthesis Method	Orientation	Crystallinity	Substrate	Thickness	Defect	Refs.
Solvothermal synthesis	**Conventional preparation:** Randomly oriented powders or thin films **Substrate induction:** Orientation can be regulated to achieve highly ordered alignment	High	Single-layer graphene Alumina tubes	Typically several hundred nanometers	Relatively low	[26,27]
Interfacial synthesis	**Liquid–solid interface (LS) method**: Direct growth on solid substrates results in parallel orientation **Liquid–liquid interface (LL) method**: Disordered monomer diffusion between two phases leads to disordered orientation, but in specific solvent, like dichloromethane systems, it presents vertical orientation	Moderate	Self-supporting interface Silicon/Silicon dioxide	Micrometer-level thickness (self-supporting) Nanometer-level thickness (porous substrates)	Considerable	[52,53]
Microwave-assisted solvothermal synthesis	**Rapid crystallization characteristics**: Reactions complete within 60 min, suppressing grain boundary defects and forming highly oriented films	High	Porous α-Al_2_O_3_	Several hundred nanometers to a few micrometers	Low	[44,45]
In situ growth	**Substrate induction:** Parallel orientation can be regulated to achieve highly ordered alignment	Moderate	Anodic aluminum oxide Polyacrylonitrile (PAN) nanofiber Silicon/Silicon dioxide	Typically several hundred nanometers	Low	[50,51,54]

## Abbreviations

The following abbreviations are used in this manuscript:

Abbreviation	Definition
COF	Covalent organic framework.
MF	Microfiltration.
UF	Ultrafiltration.
NF	Nanofiltration.
RO	Reverse osmosis.
ED	Electrodialysis.
CDI	Capacitive deionization.
MD	Membrane distillation.
PV	Pervaporation.
1D	One-dimensional.
2D	Two-dimensional.
3D	Three-dimensional.
NiPc-PBBA-COF	Ni phthalocyanine 1,4-phenyl enebis(boronic acid) COF.
SLG	Single-layer graphene.
APTES	3-aminopropyltriethoxysilane.
TFB	1,3,5-triformylbenzene.
D-A	Donor–acceptor.
ITO	Indium tin oxide.
PI-NT	Polyimide.
SAIP	Scraping-assisted interface aggregation.
AICOF	Amide and imine bilinker COF.
AmCOF	Amide-linked COF.
IL–H_2_O	Ionic liquid-H_2_O.
Tp	1,3,5-triformylphloroglucinol.
Pa	P-phenylenediamine.
FPBA	4-formylphenylboronic acid.
TTA-DFP-COF	4,4′,4″-(1,3,5-triazine-2,4,6-triyl)trianiline 2,6-diformylpyridine COF.
TFB-TAPB	1,3,5-triformylbenzene tris(4-aminophenyl)amine
AAO	Anodic aluminum oxide.
PAN	Polyacrylonitrile.
OFX	Ofloxacin.
LS	Liquid–solid interface.
LL	Liquid–liquid interface.
Bpy	2,2′-bipyridine-5,5′-diamine.
Azo	4,4′-azodianiline.
Ttba	4,4′,4″-(1,3,5-triazine-2,4,6-triyl) tris(1,1′-biphenyl) trianiline.
Tta	4,4′,4″-(1,3,5-triazine-2,4,6-triyl) trianiline.
VOCs	Volatile organic compounds.
Tp-Bpy	1,3,5-triformylphloroglucinol 2,2′-bipyridine-5,5′-diamine.
Tp-Azo	1,3,5-triformylphloroglucinol 4,4′-azodianiline.
Tp-Tta	1,3,5-triformylphloroglucinol 4,4′,4″-(1,3,5-triazine-2,4,6-triyl) trianiline.
Tp-Ttba	1,3,5-triformylphloroglucinol 4,4′,4″-(1,3,5-triazine-2,4,6-triyl) tris(1,1′-biphenyl) trianiline.
PVDF	Polyvinylidene fluoride.
CPI	Crosslinked polyimide.
Dha	2,5-dihydroxyterephthalaldehyde.
TG_cl_	Triaminoguanidinium chloride.
LP-COF	Large-pore COF.
RhB	Rhodamine B.
TC	Tetracycline antibiotic.
CNFs	cellulose nanofibers
TGTf	Triaminoguanidinium hydrochloride-tetrafluoroterephthalaldehyde.
Pa-SO_3_H	2,5-diaminobenzenesulfonic acid.
Bth	Benzene-1,3,5-tricarbohydrazide.
Tma	2,4,6-trimethoxybenzene-1,3,5-tricarbaldehyde.
Dma	2,5-dimethoxyterephthalaldehyde.
MMMs	Mixed matrix membranes.
